# Bioenergy grass feedstock: current options and prospects for trait improvement using emerging genetic, genomic, and systems biology toolkits

**DOI:** 10.1186/1754-6834-5-80

**Published:** 2012-11-02

**Authors:** Frank Alex Feltus, Joshua P Vandenbrink

**Affiliations:** 1 Department of Genetics & Biochemistry, Clemson University, 105 Collings Street. BRC #302C, Clemson, SC, 29634, USA

**Keywords:** Bioenergy grass, Genetic improvement, Bioenergy traits

## Abstract

For lignocellulosic bioenergy to become a viable alternative to traditional energy production methods, rapid increases in conversion efficiency and biomass yield must be achieved. Increased productivity in bioenergy production can be achieved through concomitant gains in processing efficiency as well as genetic improvement of feedstock that have the potential for bioenergy production at an industrial scale. The purpose of this review is to explore the genetic and genomic resource landscape for the improvement of a specific bioenergy feedstock group, the C4 *bioenergy grasses*. First, bioenergy grass feedstock traits relevant to *biochemical conversion* are examined. Then we outline genetic resources available bioenergy grasses for mapping bioenergy traits to DNA markers and genes. This is followed by a discussion of genomic tools and how they can be applied to understanding bioenergy grass feedstock trait genetic mechanisms leading to further improvement opportunities.

## Introduction

Paleobioenergy obtained from coal, natural gas and oil deposits has allowed mankind to implement unprecedented technological advances in the last 250 years. Clearly, fossil fuels will not go away any time soon, but they are a finite resource with a viable lifespan affected by rapid population expansion (7 billion+; [1]) and the threat of the further elevation of greenhouse gases on our ability to respond to unpredictable variations in climate [2,3]. While the urgency for renewable energy sources to supplant fossil fuels on a massive scale is debatable, the need for alternative energy sources is evident. Bioenergy obtained from renewable plant material is an excellent component to any alternative energy portfolio.

Bioenergy feedstock selection is dependent upon many economic factors including land use constraints [4] and impact on other non-energy commodities [5], both of which could be addressed through public policy. Other feedstock factors can be addressed via rational existing feedstock selection as well as improvement through plant breeding and genetic modification. These factors include energy density [6] and yield, cultivation costs [6], transportation logistics [7], pre-processing requirements [7], and conversion process [8]. For example, the scale-up of fermentable corn biomass (grain) to ethanol production (1^st^ generation biofuel) in the U.S. in recent years has been successful since the conversion technology and agricultural infrastructure has matured [9]. Similarly, decades of sugarcane production in Brazil made it possible to become a net energy exporting economy [10]. Conversely, the promise of converting biomass that is recalcitrant to fermentation (lignocellulose) into viable energy products (2^nd^ generation biofuels) has yet to be realized primarily due to the lack of realistic conversion techniques [11]. Thus, there is no turn-key bioenergy lignocellulosic feedstock solution at this time, but extensive research into efficient conversion process engineering and favorable feedstock properties is well under way.

The purpose of this review is to explore the genetic and genomic resource landscape for the improvement a specific bioenergy feedstock group, the *bioenergy grasses*. We define bioenergy grasses as members of the grass family (Poaceae) that employ C4 photosynthesis and are capable of producing high biomass yield in the form of lignocellulose, fermentable juice, or fermentable grain [12]. Given their proven utility as feedstock in academic and industrial interests, we focus on resources available for five specific bioenergy grasses: *Zea mays* (maize), *Saccharum* spp. (sugarcane), *Sorghum bicolor* (sorghum), *Miscanthus* spp. (Miscanthus), and *Panicum virgatum* (switchgrass). First, we discuss which grass feedstock traits are relevant to bioenergy production with a focus on biochemical conversion. Next, we discuss genetic resources available for the five bioenergy grasses to map bioenergy traits to genes. Then, we discuss genomic tools and how they can be applied to understanding bioenergy grass feedstock trait genetic mechanisms leading to further improvement opportunities. Finally, we will make the case for how modern genetic, genomic, and systems biology approaches can be coupled with bioprocessing constraints (industrial phenotypes) to breed feedstock varieties tailored to an industrial application.

## Relevant bioenergy grass traits

There are many extant bioenergy grass feedstock varieties (genotypes), which are sufficient for select conversion processes. For example, specific maize and sugarcane genotypes have been successful bioenergy grass feedstocks since high-yielding genotypes (grain and juice, respectively) have been grown at large scale for decades, and the conversion process (yeast fermentation) is well understood at the industrial level. Recent attention has been given to the more difficult problem of 2^nd^ generation lignocellulose biomass conversion into profitable bioenergy products, which has the potential for accessing the photosynthate locked into the plant cell wall for conversion into useful products. Clearly, 2^nd^ generation genotypes that produce high dry weight yields are of paramount importance, which is the opposite direction of the Green Revolution which led to small plants with high grain yield [13]. However, the identification and improvement of bioenergy grass genotypes with high biomass that efficiently respond to a given conversion process is ideal.

While there is much potential for bioenergy grasses as feedstock into *thermal conversion* processes (e.g. combustion, torrefaction, pyrolysis, and gasification), in this section we explore traits relevant to lignocellulose *biochemical conversion* processes which convert biomass into fermentable products through enzymatic hydrolysis (saccharification) [11]. The bioenergy grass feedstock traits that underlie conversion efficiency are being elucidated opening the door to genetic enhancement from existing feedstock.

### Cellulase inhibition

Cellulase enzyme cost is estimated to be ~50% of the total cost of the commercial hydrolysis process [14]. In addition, the enzymatic hydrolysis of lignocellulosic material experiences a reduction in activity over time. This reduction in activity has been attributed to hydrolysis inhibition (end product and other [15-18]), reduction in easily accessible cellulose (e.g. crystalline vs. amorphous cellulose [19]), and reduction in efficient enzyme adsorption. Increasing enzyme accessibility to cellulose has been shown to play a crucial role in improving enzymatic hydrolysis [20-24]. Finding efficient means to increase enzymatic hydrolysis is vital to the success of lignocellulosic bioenergy production.

Chemical inhibition of cellulase reduces the total amount of reducing sugar produced for fermentation. High concentrations of end-products have been known to cause a reduction in cellulase activity. For example, while cellobiose is often a product of cellulases, it has also been shown to be a significant inhibitor of the activity of some cellulase [25]. This inhibition has been shown to be reduced by supplementing β-glucosidase to cellulase solutions lacking sufficient β-glucosidase activity [26]. End-product inhibition by glucose has been shown to inhibit late stage hydrolysis rates [27-29]. In addition to cellobiose, glucose has been shown to inhibit cellulase activity in cellulases derived from *Trichoderma* species [30,31]. However, inhibitory effects of glucose do not appear to affect *Aspergillus* species to the same degree [32-35]. This often leads to *Trichoderma* cellulases being supplemented with *Aspergillus* β-glucosidase to increase saccharification efficiency on an industrial level [36,37]. Additionally, xylose and arabinose, which are produced during the hydrolysis of hemicellulose, have been shown to inhibit cellulase activity [18,38]. Substrate inhibition of cellulases has led to simultaneous saccharification and fermentation (SSF) systems becoming popular, alleviating end-product inhibition.

In addition to end-product inhibition, metal ions have been shown to be inhibitory to cellulase hydrolysis reactions. It is suggested that the Fe(II) and Cu(II) oxidize the reducing ends of cellulose, inhibiting the exo-cellulolytic activity of cellulase [39-43]. However, not all metal ions cause an inhibitory effect on hydrolysis. Kim *et al.* found that while Hg^++^, Cu^++^ and Pb^++^ caused decrease in the production of total reducing sugars, other metal ions (Mn^++^, Ba^++^, and Ca^++^) caused an increase in the total production of reducing sugars, indicating a stimulating effect on hydrolysis [44]. Two of these ions (Hg^++^ and Mn^++^) were shown to play a direct role in enzyme adsorption. Additionally, Mg^++^ was shown to stimulate the activity of glucanase from *Bacillus cellulyticus*[45]. The activity of cellulase produced from *Chaetomium thermophilum* was shown to be increased by Na^+^, K^+^ and Ca^++^, but inhibited by Hg^++^, Zn^++^, Ag^+^, Mn^++^, Ba^++^, Fe^++^, Cu^++^, and Mg^++^[46]. This indicates that metal ions play an important role in enzyme efficacy during hydrolysis, and that knowledge of the correct ratio of metal ions is essential to increasing hydrolysis activity.

Phenolic compounds are also known to inhibit cellulolytic enzymes. These phenolics are often found in lignin, and are released (as well as their derivatives) during pretreatment processes. The types of phenolics present depends largely on the composition of biomass in combination with the type of pretreatment method employed [47-49]. A variety of released phenolic compounds have been identified during chemical pretreatment of lignocellulosic biomass [50-52], which have been shown to inhibit conversion of carbohydrates into ethanol as well as to inhibit cellulase activity [38,53-56]. Cellulases, hemicellulases, and β-glucosidase enzymes have all been shown to be inhibited by these phenolic compounds [54,56-59]. The magnitude of inhibition may specific to enzyme source as *Aspergillus niger* β-glucosidase was shown to be more resilient to phenolic inhibition when compared to *Trichoderma reesei* β-glucosidase, requiring a 4x higher concentration for inhibition [38]. Introduction of tannic acid degrading enzymes (Tannases) has been shown to increase enzymatic hydrolysis, likely by reducing tannic acid’s propensity to interact and inhibit cellulase [60]. Additionally, polyethylene glycol has been shown to reduce inhibition of cellulase by tannins [61] by breaking up tannin-protein complexes. Tween 80 and PEG-4000 have been shown to prevent inhibition of β-glucosidase by reducing the tannins ability to bind the cellulase protein [61,62]. Finding additional methods to reduce the role of inhibitors in enzymatic hydrolysis is an important factor in increasing hydrolysis efficiency and profitability. Reducing the process-specific release of cellulase inhibitors through tailored feedstock genotypes is an attractive approach to enhancing enzymatic hydrolysis.

### Cellulose accessibility

Lignocellulosic material is a complex matrix of cellulose, hemicellulose and lignin [63,64]. In un-pretreated lignocellulosic samples, only a fraction of the cellulose is accessible to enzymatic hydrolysis, while the rest of the exposed biomass is lignin and hemicellulose. In order to increase access to cellulose, pretreatment methods are employed that aim to remove the lignin and hemicellulose fraction and leave cellulose available for hydrolysis. In addition, phenolic compounds such as ferulate play an important role in crosslinking lignin within the cell wall (see reviews [65-70]) and have the potential to be genetically modified to aid in the removal of specific cell wall components. There are many grass-specific features of the cell wall which have the potential to be exploited for increased bioenergy production [71]. For example, the composition of grass lignin is composed of syringyl (S), guaiacyl (G) and p- hydroxyphenyl (H) subunits that when present in varying ratios may lead to increased digestibility [68]. However, debate remains involving the role of lignin subunits in conversion efficiency [72-75].

Removal of structural components such as hemicellulose via dilute sulfuric acid pretreatment has been shown to increase accessibility to cellulose for enzymatic hydrolysis [76]. Removal of hemicellulose has been reported to increase pore volume and surface area further increasing the accessibility of cellulase [21]. Drying lignocellulosic substrates after chemical pretreatment results in the collapse of the newly formed pores, resulting in a decrease in enzymatic hydrolysis rate through reduction in available cellulose for hydrolysis [24,77]. Another pretreatment strategy which uses ionic liquids on switchgrass was shown to increase the porosity by over 30 fold, greatly increasing the accessibility of cellulose to enzymatic digestion [78]. This indicates that pore size and volume may play a significant role in increasing the rate of enzymatic hydrolysis. The identification of bioenergy grass feedstock genotypes that respond favorably to chemical pretreatment can increase end-product yield.

Lignin has been shown to play a large role in enzymatic conversion efficiency [79]. In *Miscanthus sinesens*, Yoshida *et al.* showed that removal of lignin via sodium chlorite resulted in an increase in enzymatic hydrolysis rate [80]. Yoshida *et al.* further demonstrated that the addition of hemicellulases resulted in an increase in overall hydrolysis rate, indicating that hemicellulose is an additional inhibitor of cellulose hydrolysis rates [80]. Zhao *et al.* also reported an increase in the enzymatic hydrolysis rate of sugarcane bagasse after the removal of lignin with paracetic acid [81]. Dissolution of lignocellulosic material with ionic liquid has been shown to increase enzymatic hydrolysis rates in wheat straw [82], corn stover [83] and switchgrass [78]. Kimon *et al.* showed that disolving lignocellulosic material in ionic liquid at temperatures >150°C has a large effect on saccharification of sugarcane bagasse [84]. Additionally ionic liquid pretreatment of switchgrass was shown to increase hydrolysis kinetics by over 39 fold over untreated switchgrass [78]. Ionic liquid pretreatment has also been shown to break inter and intra-molecular hydrogen bonding between cellulose strands causing an increase in the removal of amorphous components (lignin, hemicellulose) as well as an increase in surface area for cellulase adsorption [85]. These methods were both shown to superiorly increase hydrolysis rates when compared to traditional methods (dilute acid and ammonium hydroxide, respectivley). Singh *et al.* reported that ionic liquid caused disruption of the inter and intra-molecular hydrogen bonding between lignin and cellulose which initially causes swelling of the plant cell wall followed by complete dissolution [86]. Organosolv pretreatment of switchgrass was shown to preferentially remove both lignin and hemicelluloses, leaving a larger cellulose fraction which resulted in an increase in the enzymatic hydrolysis rate [87]. Rollin *et al.* showed that treating switchgrass with organozolv resulted in a similar increase in the surface area causing increased cellulase adsorption [88]. It is important to note that the promising field of ionic liquid pretreatment it still in its infancy. The current high costs of ionic liquid pretreatment limits its application to industrial scale-up, and like enzyme costs, must be reduced in order to be economically feasible on a large scale.

In addition to chemical pretreatment, naturally occurring mutations found in grasses have been shown to increase the rate of enzymatic hydrolysis via reductions in lignin. Brown midrib (*bmr*) is a phenotype found in grasses (maize [89], sorghum [90] and pearl millet [91]) that is associated with a mutation in genes involved in monolignol biosynthesis. These mutations have been shown to lead to a reduction in the total lignin content of the plant [92,93]. The brown colored midrib of the leaf has been shown to associate with a mutation in cinnamyl-alcohol dehydrogenase (CAD), which causes incorporation of cinnamyl-aldehydes in place of cinnamyl-alcohol during lignin biosynthesis [72,94,95]. Additional *bmr* varieties have been shown to have mutation in caffeic acid O-methyltransferase (COMT) [96-98]. However, both CAD and COMT mutants only exhibit reduced monolignol biosynthesis as opposed to total cessation of monolignol biosynthesis, indicating that other CAD and COMT genes may individually override complete cessation of monolignol biosynthesis. Theerarattananoon *et al.* found that a *bmr* mutant sorghum variety had less total lignin than forage, grain, sweet and photoperiod sensitive sorghum varieties [99]. In addition to lower lignin contents, *bmr* varieties have been shown to have increased susceptibility to chemical pretreatments. In sorghum, it was found that *bmr* mutants were more susceptible to alkaline pretreatment than non-*bmr* varieties [100]. Corredor *et al.* demonstrated that *bmr* sorghum varieties had a 79% hexose yield after enzymatic hydrolysis, which was higher than two non-*bmr* varieties which yielded 43% and 48% [101]. Additionally, sorghum varieties that contain both the mutations in COMT and CAD have been shown to have lower lignin contents than either mutant individually [102]. It is possible that there are additional genes and alleles leading to lowered lignin or other traits associated with higher hydrolysis rates. The identification of new as well as known lignification genes could lead to novel breeding programs where stacking of genes could result in intrinsic increases in lignocellulosic digestibility.

It is important to note that some maize *bmr* varieties have been characterized as being susceptible to lodging [103]. However, these susceptibilities were not seen in other maize studies which may be attributed to differences in genetic background [104,105]. This suggests that selecting an optimal genotype for the *bmr* mutation may be important in creating a superior feedstock. In addition to lodging, *bmr* mutants have been labeled as more susceptible to disease and pathogen attack due to reduction in the lignin barrier. However, accumulation of lignin precursors has been shown to prevent the production of virulence factors as well as limit fungal pathogens [106-108]. It has also been widely reported that *bmr* varieties experience a decrease in yield associated with reduced lignin content. This has been seen in maize [104,109,110] and sorghum [111,112]*bmr* varieties. However, sorghum *bmr* hybrid varieties have been created that experience yields similar to wild type [113], suggesting that the genetic background of the mutant variety is important in overcoming yield reduction.

Transgenic approaches have already shown potential to increase saccharification efficiency in grasses. Overexpression of miR156, which suppresses SQUAMOSA PROMOTER BINDING PROTEIN LIKE (SPL) genes, in switchgrass caused an increase in overall biomass accumulation coupled with an increase in conversion efficiency of 24.2% – 155.5% in non-pretreated lignocellulosic material and between 40.7%–72.3% increase in acid pretreated samples [114]. In addition, moderate overexpression of miR156 caused switchgrass plants not to flower, reducing the possibility of transgenic gene escape. However, it should be noted that overexpression of miR156 caused dwarfism in both rice [115] and maize [116], which greatly reduces the plants value as a bioenergy feedstock. In addition, overexpression of R3R3-MYB4 transcription factors has been shown to repress lignin biosynthesis in several species [117-120]. In switchgrass, overexpression of PvMYB4 resulted in a three-fold increase in hydrolysis efficiency [121]. However, like the overexpression of miR156, these plants experienced a smaller stature than control varieties, limiting the gains made from increased hydrolysis efficiency. Clearly, the identification of active small RNA regulatory genes that do not affect biomass yield using genomic approaches is an exciting avenue towards bioenergy grass improvement.

### Crystallinity index

Crystallinity index (CI) is a parameter that is used to determine the relative amount of crystalline cellulose in lignocellulosic material. Increased crystallinity of cellulose causes reduction in cellulase binding to cellulose due to reduced surface area. Conversely, increased amorphous cellulose causes an increase in the surface area, causing an increase in hydrolysis rates. CI has been measured using x-ray diffraction [122], solid-state ^13^C NMR [123], infrared spectroscopy (IR) [124-126] and Raman spectroscopy [127]. CI has been shown to be correlated with enzymatic hydrolysis of lignocellulosic material. In *Sorghum bicolor,* CI has been shown to be negatively correlated with hydrolysis rate in whole plant tissue [128]. It has also been shown in sorghum as well as maize that stem has a higher crystalline content than leaf tissue [129]. Furthermore, sorghum *bmr* mutants as well as wild type varieties experience an increase in CI after pretreatment with 1M NaOH. This observation is attributed to the removal of the amorphous component of the lignocellulosic biomass, leaving a larger fraction of crystalline material. However, it was also observed that an increase in the concentration of NaOH to 5M showed a decrease in CI, which was attributed to the crystal structure change and cellulose amorphization [100]. A similar trend was seen in dilute acid pretreatment of five sorghum varieties. Dilute acid pretreatment of sorghum at 140°C resulted in an increase in CI, however increasing the temperature during pretreatment to 165°C resulted in a decrease in the CI of 4 of 5 sorghum varieties [99]. This change in cellulose composition after pretreatment has been previously demonstrated in various industrial cellulose samples pretreated with NaOH [130,131]. Sugarcane bagasse was also shown to experience an increase in crystallinity after pretreatment with peracetic acid, which was attributed to a decrease in the amorphous component of the plant biomass [81]. Corredor *et al.* demonstrated dilute acid pretreatment of *bmr* and non-*bmr* sorghum varieties were shown to increase CI after pretreatment [101]. In addition, hydrolysis of the same samples resulted in a reduction in CI. Liu *et al.* found that like sorghum, acid pretreatment of maize biomass causes an increase in CI. However, the harshest pretreatment conditions cause a decrease in crystallinity, likely due to disruption of the cellulose crystalline structure [132]. This trend was confirmed by Mittal *et al.*, who also demonstrated that crystallinity of corn stover depends on specific conditions of alkali pretreatment. Additionally, Barl *et al.* demonstrated that maize husks experienced an increase in CI after both acid (H_2_SO_4_) and alkali (NaOH) pretreatment processes [133]. It should be noted that previous studies have demonstrated that the cellulose binding domain of cellulases disrupt cellulose crystalline structure and causes a decrease in CI [134,135]. This suggests that cellulose binding plays a role in conjunction with a decrease in cellulose content in the reduction in crystallinity index during enzymatic hydrolysis. Therefore, finding favorable genetic variation in endogenous and pretreated CI is a logical approach to improve hydrolysis yield [128].

Not all pretreatment strategies lead to an increase in CI. Pretreatment strategies that are particularly harsh initially increase CI through removal of amorphous components, followed by subsequent dissolution of crystalline cellulose. For example, Kimon *et al.* demonstrated that dissolving sugarcane lignocellulosic material with ionic liquids at temperatures >150°C causes a reduction in the cellulose CI and a large increase in glucan saccharification, while temperatures <150°C has a small effect on crystallinity, which was associated with a slower initial rate of glucan saccharification [84]. Therefore, a screen for bioenergy grass genotypes that respond to harsh pretreatments in a favorable way could identify better feedstocks.

CI has been shown to differ between plant species, as well as different varieties within a species. When compared to different sorghum varieties, maize has been shown to have a higher CI [99]. Vandenbrink *et al.* demonstrated that CI differed between 18 different varieties of *Sorghum bicolor,* and these differences in CI were associated with hydrolysis rate [128]. Harris *et al.* found that crystallinity index differed among a large variety of plants which included sweet sorghum, switchgrass, giant *Miscanthus*, sweet *Miscanthus*, flame *Miscanthus*, gamagrass, big bluestem and *Arabidopsis*[136]. However, it must be pointed out that many of these species were only tested on a small number of varieties, which may not give an accurate depiction of CI in a diverse population where one genotype is one data point. These studies provide evidence that due to differences in CI between species and variety, there may be a significant genetic component that is associated with the trait.

There is much debate about the changes in crystallinity experienced during enzymatic hydrolysis of lignocellulosic materials. Various studies have demonstrated that amorphous cellulose components are hydrolyzed preferentially to crystalline components, resulting in an increase in crystallinity as enzymatic hydrolysis occurs [80,137,138]. However, various other studies have demonstrated that hydrolysis results in little change to crystallinity over the course of enzymatic hydrolysis [139,140], which was attributed to the synergistic action of *endo* and *exo*-glucanase activities [87,141]. However, it should be noted that studies have shown that the cellulose binding domain of multiple cellulases disrupt the supermolecular structure of cellulose, resulting in a decrease in CI [134,135]. This creates a difficult task in measuring changes in CI during enzymatic hydrolysis.

### Enzyme adsorption

Non-specific cellulase adsorption to biomass plays a crucial role in determining the effectiveness of enzymatic hydrolysis. Due to the high cost of enzymes for commercial scale hydrolysis, adsorption and desorption rates in specific genotypes should be pre-determined. After hydrolysis, enzymes can either remain adsorbed to the substrate or unbound in the hydrolysate [142]. Cellulase adsorption depends largely on the concentration of the protein, as well as cellulase concentration and available surface area [143]. Initial protein adsorption has been shown to correlate with the initial rate of cellulose hydrolysis [19,144]. Multiple studies have shown that total enzyme adsorption is directly related to hydrolysis rate and yield [145-148]. Strong correlations between available surface area and rate of hydrolysis have also been observed [23,149,150]. This increase in hydrolysis rate can be attributed to increased adsorption. Nutor *et al.* found that initial protein adsorption occurs quickly, reaching a maximum in 30 minutes, followed by 55-75% desorption [151]. Increasing the amount of enzyme adsorbed onto cellulose substrate is a potential avenue to increase hydrolysis rates, and it remains untested if specific cellulases are better adsorbed in specific bioenergy grass feedstock varieties.

Cellulase adsorption to lignin reduces cellulase activity by sequestering the enzyme away from its substrate. After the completion of hydrolysis, non-specific binding to lignin that has been freed during hydrolysis has been shown to occur, where 30-60% remains bound to the lignin fraction [152,153]. This non-specific binding has been shown to be only partly reversible [154]. Adsorption of cellulases to isolated lignin has been reported, supporting claims that non-specific binding occurs to the lignin fraction during hydrolysis [155,156]. Any cellulase bound to lignin is not available to hydrolyze cellulose, limiting overall efficiency. Hydrolysis rates of cellulose has been shown to be correlated with the tightness and affinity of adsorption [157]. Removal of lignin does not only reduce the steric hindrance to the enzyme, but also reduces the lignin available for non-specific binding [158,159].

Protein adsorption interactions are usually non-covalent (hydrogen bonding, electrostatic or hydrophobic interactions [160]). Surface characteristics of lignocellulosic material are thought to play a major role in cellulase adsorption where the high surface area hydrophobicity results in increased adsorption. Cellulases have been shown to have hydrophobic amino acids exposed on the outside of the protein, which interact with the hydrophobic surface of cellulase [161]. The affinity of cellulase for hydrophobic substrates may explain non-specific binding to lignin which is highly hydrophobic. In addition to this, metal ions have been shown to increase (in the case of Mn^++^) and decrease (in the case of Hg^++^) the adsorption affinity and tightness of binding to the hydrophobic surface of cellulose [44].

In order to drive down the cost of enzymatic hydrolysis, strategies to recycle cellulases are being developed [141,162-165]. Enzymes can be recovered from either bound substrate or from the liquid hydrolysate that remains after the first round of hydrolysis. Recovery of the enzyme from bound substrate can be achieved through washing with surfactant (such as Tween 20 [166]) or through recovery of the solid substrate in which the cellulase remains bound [162]. Use of cellulase recovered from lignocellulosic residue for subsequent rounds of hydrolysis have been shown to experience reduced activity, which has been attributed to accumulation of bound lignin after each successive round of hydrolysis [154,163]. Recovery of enzyme from the liquid hydrolysate has been traditionally been done through ultracentrifugation techniques [142,167,168]. While this method has been proven effective, it would be costly to scale up to industrial magnitudes. A more effective method may be to exploit cellulase affinity for cellulose, in which the addition of cellulose to cellulase-containing hydrolysate results in re-adsorption onto the fresh cellulose substrate [163,169,170]. Tu *et al.* found that addition of fresh substrate to hydrolysate recovered ~50% of cellulases [171]. Additionally, bound enzyme was shown to be able to be recovered by contacting the bound substrate with fresh substrate [172]. However, sequential hydrolysis with recovered enzyme results in decreasing hydrolysis rates due to non-specific binding. Additionally it must be noted that β-glucosidase does not bind to cellulose substrate, and must be added at the beginning of each round of hydrolysis in order to prevent the buildup of cellobiose and the resulting substrate inhibition [171]. It is therefore necessary to develop techniques that are able to efficiently desorb cellulase from bound substrate. Deshpande *et al.* found that 90% of cellulase was recoverable from steam-exploded wheat straw [152]. Jackson *et al.* found that using a surfactant such as Tween 80 resulted in a recover of 6 – 77%, depending on concentration of Tween 80 and pH of the solution [166]. Additionally, Jackson *et al.* revealed that the highest protein recovery does not necessarily dictate the highest activity recovery, and that alkali conditions may be responsible for deactivation of the enzyme. Otter *et al.* demonstrated that Tween 80 and Triton X were able to desorb 65-68% of bound cellulase under alkaline conditions [173]. Qi *et al.* demonstrated that enzyme recycling of alkali and dilute-acid wheat straw was comparable when using ultracentrifugation and additional substrate techniques [174]. However, the additional substrate technique requires addition of β-glucosidase after each round of hydrolysis, whereas ultracentrifugation does not. Finally, there was a noticeable difference in enzyme recovery between dilute-acid and alkali pretreated samples, where alkali pretreated samples were able to desorb a larger amount of cellulase. While this discussion is focused on the putative industrial processes, it may be that specific feedstock varieties naturally exhibit lower adsorption rates that would further enhance the engineering endeavors.

In order for bioenergy to become a sustainable alternative to traditional fossil-fuel based transportation fuels, significant improvements to current enzymatic hydrolysis methods must be made. Reduced enzyme activity has been shown to be related to end-product inhibition, production of phenolic compounds from lignin, as well as metal ion inhibition. Additionally the reduction in easily accessible cellulose through steric hindrance and high crystalline to amorphous cellulose levels cause a reduction in cellulose available for enzymatic hydrolysis. Non-specific binding of cellulases to solubilized lignin has also been associated with reduced hydrolysis rates. Finally, adsorption has been shown to be correlated with the initial rate of hydrolysis, while enzyme desorption is essential for enzyme recycling and reducing the cost of enzymes in bioenergy production. While these process components are being examined at the engineering level, a simple screen of existing bioenergy grass varieties could identify genotypes with a favorable trait baseline making the process engineering task less difficult.

## Bioenergy grass genetic mapping resources

There are tens of thousands of bioenergy grass genotypes in seed banks that have yet to be screened for favorable bioenergy traits. In fact, many traits that have been shown to deeply impact bioconversion yields have only been tested in a handful of genotypes. Surely, there are a multitude of relevant traits yet to be discovered. Therefore, we believe that genetic improvement is often premature until all screening options have been exhausted. With this caveat, genetic improvement in bioenergy grass feedstock can be achieved through transgenic manipulation or plant breeding programs. For example, centuries of selection have led to crops that provide high grain yields ideal for food production [13,175]. Many “elite” cultivars are dwarf varieties that allocate photosynthate towards larger grain yields as opposed to high cellulosic biomass. In grasses, the trend towards reduced lignocellulosic biomass could be rapidly reversed as genetic loci for plant height are few and well characterized [176-178]. In addition, the bioenergy traits discussed above can be genetically mapped to genomes, DNA markers associated with the trait developed, and alleles sorted into elite and novel cultivars. Once relevant DNA markers are identified, these traits can be selected for in breeding programs using marker assisted selection (MAS; [179]) or genome selection (GS; [180]) techniques. If the causal gene is identified, it can be introduced transgenically [181] to create elite bioenergy feedstock varieties.

In this section, we discuss the extensive genetic tools available for mapping traits in the genomes of bioenergy grasses as well as examples of previously mapped bioenergy traits.

Genetic mapping techniques available for bioenergy grasses include mapping Quantitative Trait Loci (QTLs) through linkage mapping in biparental populations [182], association mapping in a genetically diverse population [183], and nested association mapping (NAM) [184,185]. QTL mapping requires relatively sparse marker coverage but identifies broad chromosomal regions associated with a trait of interest [182]. Association mapping analysis often requires prior knowledge of genes of interest or a full genome scan with high marker coverage to be successful [186]. Nested association mapping (NAM) populations exploit the benefits of both QTL and association mapping approaches [184,185]. It should be noted that genetic population structure can cause confounding correlation between markers and phenotypes within subpopulations [187,188]. The existence of distinct subpopulations can cause bias in the estimation of allelic effects and errors in QTL detection [189]. Thus, it is critical to generate panels that are genetically diverse and where population structure is clarified and corrected prior to genotype-phenotype associations [190]. All three genetic resources exist for diploid maize and sorghum bioenergy grasses and have been successful in mapping traits for years (see examples below). These approaches are more difficult in complex polyploids such as switchgrass, *Miscanthus*, and sugarcane, but there has been success in QTL mapping for these species (see examples below).

### Quantitative trait loci

Genetically defined mapping populations are a useful resource for locating DNA markers and mapping genes associated with desirable bioenergy traits. In these populations, quantitative trait loci (QTLs), intervals in the genome where DNA markers show a non-random association with a quantitative trait, can be identified [191], and the causal gene can possibly be mapped, albeit with difficulty (but see below). DNA markers associated with bioenergy QTLs can be used to breed superior varieties without extensive phenotyping [179] that contain a collection of genes desirable in a bioenergy feedstock. A key advantage of QTL mapping is that polymorphic DNA markers can be easily developed without a reference genome and they do not need be at high density across the genome.

In the diploid species sorghum, QTLs have been identified for many potentially advantageous genes valuable to biofuel production. QTLs related to leaf size including leaf width and leaf length [192] as well as leaf yield and composition [193] have been identified. Stem morphological traits such as height [178,193-203], diameter [192] and tillering characteristics [191,193,195,202] as well as stem composition and sugar content [193,201] have been associated with QTLs in sorghum. In addition, QTLs for flowering time or maturity have been shown to increase overall biomass by increasing the period of plant growth [178,194-198,201-205]. QTLs have also been analyzed for kernel weight [191,194,195,199,200,206,207] as well as grain composition [200,206,208,209]. In addition, QTLs for post-harvest regrowth (ratooning) [191,193] may have the potential to increase total biomass yield producing additional biomass post-harvest. A recent study has mapped bioenergy QTLs, including biomass and stem sugar content, in a cross between a grain and sweet sorghum, [210]. The DNA markers identified in these studies can be used in breeding programs and demonstrate that markers for novel bioenergy traits such as the traits described above can easily be generated in existing or novel QTL mapping populations.

In maize, extensive research into QTLs of agronomic traits has been conducted. QTLs for forage quality and biomass composition have been comprehensively studied [211-219] and may have the potential to increase conversion efficiency. Also, because corn is a major food crop, thorough investigation of mapping populations has been conducted leading to the identification of a multitude of grain yield QTLs [220-233] which may lead to larger starch-derived ethanol yields. Additionally, QTLs for biomass related traits including both plant height [177,234-242] and plant maturity/flowering time [234-240,243,244] have been characterized, which could lead to increases in overall biomass yield. Leaf biomass characteristic QTLs [236,245-247] have also been identified which can lead to increased biomass as well as increased crop density resulting in greater yields. As with sorghum QTL studies, the maize mapping populations used in these studies can be used to map additional bioenergy traits and these DNA markers can be used in selection programs.

Complex polyploids such as *Miscanthus sinensis,* switchgrass, and sugarcane have had substantially fewer QTLs identified relative to the diploid grasses: sorghum and maize. In *Miscanthus*, plant biomass including leaf yield, stem yield and total plant height have been identified [248,249] leading to potential increases in total biomass. Additionally, flowering time QTLs have been identified which may lead to increased biomass accumulation [250,251]. *Miscanthus* also has potential as an energy source for thermal conversion. This has led to the identification of QTLs that effect thermal conversion efficiency [252,253]. To date, there have not been QTLs identified for the composition of *Miscanthus* biomass or forage quality, but the extant mapping populations are an excellent resource for mapping these traits. In sugarcane QTLs for stem sugar content have been identified [254-257], but few other bioenergy QTLs have been identified. These representative studies demonstrate that QTL mapping is a realistic tool for mapping complex traits in polyploid species. Below we discuss how modern sequencing techniques can be used to sequence large DNA segments underlying the QTL that becomes a powerful resource for identifying candidate genes even in complex polyploids.

QTL mapping in polyploid bioenergy grasses should improve with the development of new genomic resources. Recently, a high density genetic map has been developed for switchgrass [258], and two high resolution linkage map were created for *Miscanthus sinensis*[259,260]. These high-density maps open the door mapping QTLs to other genome through comparative genomics. For example, the *Miscanthus* map studies found that that of the sequenced grass species, sorghum, has the closest syntenous relationship to *Miscanthus* and that *Miscanthus sinensis* is of tetraploid origin consisting of two sub-genomes. These genetic maps will allow researchers to translate genetic tools from sorghum such as QTL studies and a sequenced genome via synteny relationships, thereby expanding the toolkit available for *Miscanthus*. In addition, the high density linkage maps can be used for *Miscanthus* genome assembly as well as QTL studies. Known and as yet undetected QTLs are a valuable method to identify DNA markers, often in multiple genome positions, that can be used to select for improved feedstock varieties before a crop development cycle is complete.

Minimal progress has been made in the development of superior cultivars from the identification of QTL associated with bioenergy traits. This may be due to the limitations in the transferability of QTL information due to QTLs being specific to alleles from inbred mapping parents. It may be that robust QTLs detected under multiple genetic backgrounds will be required. However, MAS stacking of QTLs (pyramiding) has been successful in other plant species as an avenue of crop improvement. Zhang et al. used QTL pyramiding to increase downy mildew tolerance in wild lettuce (*Lactuca saligna)*[261]*.* In another example, rice yield [262] as well as grain size and shape [263] have been modified through QTL pyramiding strategies. This suggests that given the ideal genetic background, genetic improvement of bioenergy crops through QTL pyramiding may be a viable way to produce superior feedstocks.

The NAM method for mapping QTLs relies on selection of a genetically diverse founding population which is derived from a common parent to create a large population of related progeny (often in the form of Recombinant Inbred Lines or RILs). NAM has the benefit of providing high QTL mapping resolution without requiring high marker density within the population [264]. In maize, a NAM population was created consisting of one common parent crossed with 25 diverse parents to produce 5,000 genetically distinct offspring [264]. A sorghum NAM population is under development [265]. QTLs for leaf architecture (including leaf angle, leaf length and leaf width) have been identified using the maize NAM population [185]. In addition, NAM has been used to identify QTLs for complex traits such as resistance to northern leaf blight in maize [266]. While NAM incorporates high resolution QTL mapping with low marker coverage and high heterogeneity, it also requires large population size and a structured population in order to be informative. This technique also requires the screening of a large number of individuals, which makes the identification of complex phenotypes potentially very labor intensive. However, the NAM and other advanced genetic approaches are a powerful approach to dissect the genetic architecture of complex bioenergy traits.

While QTL studies have potential for bioenergy gene discovery, they also have limitations. Due to genetic heterogeneity, QTLs may be overestimated or not detected. There are also a variety of problems that arise in QTL mapping of polyploid genomes such as sugarcane and *Miscanthus*. These include increases in the number of genotypes per marker or QTL due to the increased number of chromosomes in the homeologous set, the dosage of marker and QTL in the parents and progeny are not obvious or observable, additional copies of a marker can mask recombination events, and the pairing behavior of chromosomes during meiosis is usually unknown [267]. Furthermore, low density genetic maps make it difficult to locate genes within a QTL region, which can contain thousands of genes. Dense genetic maps based upon sequence tagged markers, as is the case for sorghum [268], are readily mapped to other genomes. In this way, bioenergy QTLs can be identified in diploid sorghum and mapped to complex genome bioenergy grasses for causal gene inference and validation.

### Association mapping (diversity) panels

Association mapping is an alternative method for mapping QTLs that is based on linkage disequilibrium (LD) occurring from historical recombination events in genetically diverse populations [269,270]. Association mapping utilizes marker-phenotype associations to determine if certain DNA markers co-segregate with a phenotype of interest. Association mapping generally falls into one of two categories: i) candidate gene association mapping, which looks for markers and causal variation in a subset of genes that are of interest for polymorphisms and ii) genome wide scan association mapping (GWAS), which scans the whole genome using dense marker sets to find marker associations with complex traits. Association mapping offers multiple benefits over traditional QTL mapping populations. QTL mapping populations suffer from restrictions due to limited genetic heterogeneity in that a QTL that is mapped in one mapping population derived from two genetic backgrounds and may not be applicable to other populations with parents derived from different lineages [271,272]. Association mapping panels, however, benefit from having higher resolution of identified QTLs than traditional QTL mapping methods [273]. While association mapping requires a large diverse germplasm (diversity panel) of individuals to map QTLs, it does not require generation of inbred or backcrossed populations.

Association mapping populations have been created for the bioenergy crops maize [274,275], sorghum [176,276] and sugarcane [277]. In sorghum, association mapping has led to the identification of markers for height, flowering time, tiller number and stem sugar [278,279]. In maize, association mapping has led to the identification of markers for flowering time [187,280,281], kernel composition [282] as well as starch accumulation [283]. Fewer studies have been conducted in sugarcane, which has a large complex genome with high ploidy levels ranging from 5x – 14x [284]. Wei *et al.* mapped disease resistance in 154 sugarcane cultivars [277]. A key drawback to association mapping is that the large population size required for successful identification of trait markers requires that phenotyping the plants be done in a high-throughput manner which requires a large labor force or robotics. Often, this reduces the scope of DNA markers that can be identified to traits where phenotyping is less intensive.

### Reverse genetics

In addition to the forward identification of DNA markers (and genes) by mapping a bioenergy trait to a DNA polymorphism, reverse genetic tools exist for the identification of bioenergy genes from a panel of known mutants. If the mutants are created in a parent with a favorable bioenergy trait baseline, it is possible to map genes and improve feedstock at the same time. In the TILLING approach (Targeting Induced Local Lesions IN Genomes), point mutations are randomly created throughout the genome by treating seeds with a mutagen (e.g. ethyl-methanesulfonate (EMS)) [285-287]. These plants are selfed and screened for phenotypes of interest. The DNA sequences from plants with mutant phenotypes can be compared to the non-mutagenized parental DNA to determine the relevant mutation. For example, DNA can be purified in a high throughput manner [288] and sequenced using high-throughput techniques for the discovery of rare mutations [289]. If the founding parent of the TILLING population has a sequenced genome as a reference, sequencing of select mutant individuals in candidate genes or whole genome resequencing can be done to identify specific gene mutations that lead to phenotypes of interest (*e.g.*[290]). As proof of principle, a sorghum TILLING population has been effective in the discovery of mutations giving rise to the bioenergy-relevant brown mid-rib phenotype [291] and altered hydrogen cyanide potential [292]. Once the gene variant underlying a trait is identified, the gene can be sequenced (e.g. PCR amplicon sequencing), and any DNA variants tested for association in additional genotypes from the source and related organisms.

TILLING populations have been created for the bioenergy crops maize [293] and sorghum [294]. TILLING has the potential to identify bioenergy traits such as flowering time, total biomass, grain yield, conversion efficiency, etc. TILLING as a strategy for biofuel improvement does have its limitations. Due to the mutations induced by EMS being distributed randomly throughout the genome, the TILLING strategy can require screening thousands of individual lines to identify mutants in a trait of interest. This requirement of high-throughput phenotyping techniques limits the throughput of mutant selection gene detection. Furthermore, polyploid genomes present problems associated with finding recessive mutants due to the number of gene copies present in the genome. In the case of bioenergy grasses, this is strong rationale for first identifying a causal genetic lesion in a diploid genome (e.g. sorghum) and then testing the effect of the mutation in more complex genomes through plant breeding or transgenics. In summary, advanced genetic and mutant populations are a powerful approach to create varieties and map genes relevant to bioenergy feedstock.

## Bioenergy grass genomic resources

The crop genetic studies reviewed above have identified DNA markers associated with some high priority bioenergy related traits such as total biomass and conversion efficiency. These biomarkers have immediate utility in bioenergy grass improvement, and it is certain that the future will reveal many more biomarkers linked to known and novel bioenergy traits. However, the DNA biomarker often merely tags DNA near the gene(s) causing the favorable phenotype. While effective in breeding, this level of information leaves the underlying casual biochemical pathways and mechanisms in the black box. If the molecular mechanisms (and specific genes) underlying a trait were to be deciphered, then the art of plant breeding could be enhanced by searching for gene variants in other genes in the same pathway(s) as the initially described causal gene. Fortunately, the genome blueprints for specific bioenergy crops have been deciphered in the last decade. Using a reference genome assembly as a guide, it is now possible to associate genetically mapped biomarkers with nearby candidate genes and their functional activities. This section surveys genomic resources available for bioenergy grasses and discusses their utility in a genetically mapped trait context.

While genome-wide measurements of gene output can be obtained and interpreted without a reference genome, a high-quality, annotated reference genome assembly provides a natural scaffold to organize and interpret genetic and genomic analyses. In the case of bioenergy grasses, three key reference genomes have been sequenced and annotated: maize [295], sorghum [296], and switchgrass (http://www.phytozome.org/panicumvirgatum.php). Once a genome assembly is constructed, it is annotated for sequence features including gene models and copy number (gene duplications), regulatory features, heterologous genome alignments (synteny), and other dynamic features such as gene expression levels under different internal and external cues. An excellent genome assembly resource for many plants, including maize, sorghum and switchgrass, can be found at the DOE-JGI Phytozome website [297].

The genome assembly sequence is a stable coordinate system to associate genome-mapped genetic signals (e.g. QTL biomarkers, trait-associated SNPs) with functional genomics information such nearby genes, gene expression levels, and biochemical pathways. If the sequences of DNA biomarkers are known, one can often locate the approximate genome position of a genetic signal and find neighboring genes in a physical context. Through the genome browser, biomarker DNA sequences can be positioned using BLAT/BLAST alignment tools or possibly through keyword searches. In some cases, biomarker positions have been pre-computed such as maize genetic markers accessible at [298]. Neighboring gene models are often annotated for function, usually via homology mapping, and provide clues that a given gene could be involved in the expression of a bioenergy trait. Gene function annotations include conserved protein domains (e.g. Interpro [299], Gene Ontology (GO) terms [300], and biochemical pathways (e.g. KEGG; [301]) including well annotated metabolic enzymes (e.g. RiceCyc at Gramene [302]). These annotation terms provide clues into what a gene near the biomarker is doing including possible pathway involvement, an indicator of gene-gene interaction and complex trait mechanism. It should be noted that genome browsers are highly dynamic and are constantly being updated with new information relevant to basic biology and possible bioenergy trait mechanisms.

While a reference genome view of an individual organism is invaluable, there are a growing number of databases focused on genome comparison and mapping function between species. This *translational genomics* approach is very important for the bioenergy grasses as gene function information can be discovered in a well-studied diploid organism such as maize, rice, and sorghum for which the genome is easier to analyze relative to complex polyploids like switchgrass, sugarcane and *Miscanthus*. Translational genomics is possible between bioenergy grasses because grass genomes in general have maintained a similar structure analogous to mammalian genomes since they diverged from a common ancestor 50–70 million years ago [303]. Therefore, genomes of non-bioenergy grasses including rice [304] and *Brachypodium*[305] are also useful reference blueprints for grass gene function discovery and genome comparison [306]. Through grass genome comparison, gene function can be inferred in a poorly studied genome by identifying orthologous chromosomal segments. For example, the VISTA comparative genome browser (http://pipeline.lbl.gov; [307]) visualizes pre-computed alignments between the genomes of maize and sorghum as well as many other plants. A rich resource for genetically mapped information and grass genome comparison is Gramene ([302,308]). Finally, the Comparative Saccharinae Genomics Resource (CSGR; [309]) is focused specifically on the grasses including and related the bioenergy grasses. For a deep study of these resources, the reader is directed to relevant chapters in [310]. The macroconservation of grass genome structure is critical for genomic translation between bioenergy grasses with complex genomes such as sugarcane, switchgrass and *Miscanthus*. It may be a long time before additional reliable assemblies of complex polyploid genomes are realized, and at this time, we suggest that sorghum is an ideal C4 bioenergy grass reference genome due a relatively small annotated genome and close evolutionary proximity to other C4 bioenergy grasses.

The genome assembly provides physical coordinates of known genes, and intergenome comparison explores the dynamic movement of genes over evolutionary time scales. A reference genome assembly is also a framework for organization dynamic gene output measurements. For example, bioenergy grass gene output at the RNA level has been measured for over a decade using 1^st^ generation genomic tools including the conversion of tissue and treatment specific RNA samples into cDNA followed by tedious cloning and sequencing. These Expressed Sequence Tags (ESTs) have proven invaluable in gene identification and can be found in databases at the National Center for Biotechnology Information (NCBI EST database) as well as the genome databases mentioned above. Massively parallel measurements of the RNA transcriptome response under multiple treatments and conditions have been made for bioenergy grasses using DNA microarrays. These experiments are stored into raw and processed forms at the NCBI Gene Expression Omnibus (GEO) database and are an excellent functional genomic data mining resource for the bioenergy grasses. For example, differences in gene expression in a genetically defined population can be associated with traits as eQTLs [311]. In addition, thousands of gene co-expression interactions can be mined from these datasets and transformed into gene interaction networks (see examples below). These functional genomics resources have been effective in understanding the molecular function of many bioenergy grass genes.

In recent years, rapid advances in DNA sequencing technology coupled with a reference genome for mapping sequences have resulted in multiple powerful next generation genomic analytical tools [312]. New sequencing technologies are capable of sequencing 10^5^-10^8^ DNA molecules in a single experiment. As opposed to measuring molecule levels through hybridization to microarrays, this depth of coverage allows for molecule counting such as RNA-derived cDNA (RNAseq) or genomic DNA (re-sequencing) fragments to such a degree that quantitative comparisons can be made between samples. Example applications include transcriptome profiling with RNAseq [313], *de novo* transcript assembly [314], single nucleotide polymorphism (SNP) discovery [315], is the discovery of rare mutations in mutagenized (e.g. TILLING) populations [289,290], genotyping by sequencing (GBS; [316]) followed by GWAS or GS [183]), as well as whole [317] or partial genome *de novo* genome assembly [318]. In short, emerging sequencing technologies provide a high resolution lens into the dynamic biology underlying organism development.

Ongoing and historical genetic studies of bioenergy traits can be the immediate beneficiaries of these new sequencing technologies in that known gene regions can be sequenced and validated. For example, given the correct mix of resources, candidate genes and QTLs can now be cloned in a cost effective manner. In one scenario, a QTL for a relevant trait is mapped even at low marker resolution without a reference genome. Then, marker probes proximal to the QTL are used to screen a BAC library to identify nearby BACs. Once candidate BACSs are identified, they can be pooled and cheaply sequenced as has been performed for melon (57 BACs; [319], the complex genome of barley (91 BACs; [320]), and cacao (27 BACs; [318]). BAC pool assemblies can be annotated for candidate genes, used to design probes for additional BAC selection, and act as a reference sequence for resequencing applications. Of course, the process of BAC selection is enhanced if a physical map exists that can be used to identify a BAC minimum tiling path (e.g. [318]). In the case of switchgrass, a physical map might resolve the polyploidy issue in BAC selection [321], so individual genomes can be separately pooled thereby reducing the probability of intergenome misassembly.

Many bioenergy traits including those outlined above are complex in that they are controlled by multiple genes. By looking at a bioenergy trait (like those discussed above) as a systems biology problem, it may be possible to identify multiple markers or causal alleles that can be mixed in an appropriate genetic background to achieve the desired effect on yield. A near complete set of genes is known for a growing number of grasses (e.g. sorghum, maize, rice), but how these genes function in concert is poorly understood. Fortunately, modern genomic tools allow for the detection of gene dependencies in the context of a relevant biochemical pathway or mapped trait that can be woven into gene interaction networks [322]. For example, gene interaction networks can be constructed that represent the non-random co-expression of transcripts between genes [323,324] or the physical interaction of gene products at the level of protein:protein interaction (PPI; [325,326]). Integrated gene sub-networks can be parsed from the overall network and non-randomly coupled with known biochemical pathways (e.g. fermentable sugar metabolism) or genetic signals (e.g. biomass yield) through a reference genome using systems biology techniques [323,327,328]. For example, gene co-expression networks have been constructed for many plants including rice [329,330] and maize [323]. Co-expressed gene modules have been identified in these networks, and some of the networks are enriched in genes that when mutated give rise to specific phenotypes that can be translated to the maize genome via conserved sub-graphs [323]. Gene regulatory networks can also be mapped to co-expressed gene modules [331]. It is possible to construct additional co-expression networks from other bioenergy grasses using RNAseq input (e.g. potato network [332]).

A systems genetics approach allows for both the prediction of complex polygenic genotype-phenotype interactions and also the ability to translate this information from diploid to polyploid genomes, a key asset in bioenergy grass improvement. We believe that gene interaction networks will significantly reduce the candidate gene list underlying a bioenergy trait if the requirement is made that interacting genetic signal genomic positions (e.g. a QTL set, multiple LD blocks from a GWAS study, or genes mapped in mutant lines that result in the same phenotype) must overlap with tightly interacting genes from the network (e.g. [323]). It is at the intersection of genetics and genomics that complex bioenergy traits, which by definition are polygenic, can be tested as a genetic sub-system as opposed to breaking the system into individual genetic components such as a single large-effect QTL.

## Conclusions

Given the uncertainties involved with long term fossil fuel production and increased carbon emissions affecting global climate, the pursuit of sustainable fuels from lignocellulosic biomass is important. We conclude that a deeper understanding of feedstock traits affecting bioconversion such as enzyme inhibition, cellulose accessibility, and enzyme adsorption will ameliorate hurdles to bioenergy production so that it is competitive with current fossil fuel based transportation fuels. While these factors limit the efficiency of enzymatic bioconversion, they also provide a myriad of opportunities for end-product yield improvement through feedstock genetics coupled with process engineering. Breeding programs that have historically focused on increased grain yields can be shifted to focus on traits yielding high-biomass, hydrolysis-efficient bioenergy crop varieties. It should be noted, however, that vast bioenergy grass seed stocks still need to be screened for high yield baselines prior to breeding new varieties. For example, future or extant varieties that contain low lignin (such as *bmr* maize, sorghum and millet) may help to reduce steric hindrance to hydrolytic enzymes as well as reduce non-specific binding and increased enzyme recovery. Additionally, reduced lignin content has potential to reduce the amount of phenolic compounds released during pretreatment and hydrolysis, which reduces inhibition to cellulase. Through the coupling of DNA biomarkers to these traits, better crops can be developed through marker-assisted selection, and rapid advances in genomic and systems biology techniques should reveal novel biochemical mechanisms that can be engineered into current feedstock varieties. It is our belief that close collaboration between the plant breeder, systems biologist, and process engineer will result in accelerated development of bioenergy grass feedstock tailored to a specific conversion process thereby increasing bioenergy viability through *industrial genetics*.

## Abbreviations

CAD: Cinnamyl-alcohol dehydrogenase; CI: Crystallinity index; COMT: Caffeic acid O-ethyltransferase; DNA: Deoxyribonucleic acid; EMS: Ethyl-methanesulfonate; GS: Genome selection; GWAS: Genome wide scan association mapping; LD: Linkage disequilibrium; MAS: Marker assisted selection; NAM: Nested association mapping; QTL: Quantitative trait loci; RIL: Recombinant inbred line; RNA: Ribonucleic acid; SNP: Single nucleotide polymorphism; TILLING: Targeting Induced Local Lesions IN Genomes.

## Competing interests

The authors declare that they have no competing interests.

## Authors’ contributions

FAF and JPV researched and wrote the manuscript. All authors read and approved the final manuscript.
